# The current management of acute uncomplicated appendicitis: should there be a change in paradigm? A systematic review of the literatures and analysis of treatment performance

**DOI:** 10.1186/s13017-017-0157-y

**Published:** 2017-10-16

**Authors:** Samuel Ho Ting Poon, Jennifer Wah Yan Lee, Ka Man NG, Gloria Wing Yan Chiu, Brian Yung Kong Wong, Chi Chung Foo, Wai Lun Law

**Affiliations:** 1 The Li Ka Shing Faculty of Medicine, The University of Hong Kong, Hong Kong, China; 2 Department of Surgery, The University of Hong Kong, Queen Mary Hospital, 102 Pokfulam Road, Hong Kong, China

**Keywords:** Appendicitis, Antibiotic therapy, Appendectomy, Non operative management

## Abstract

**Introduction:**

Appendectomy has long been the mainstay of intervention for acute appendicitis, aiming at preventing perforation, peritonitis, abscess formation and recurrence. With better understanding of the disease process, non-operative management (NOM) with antibiotics alone has been proved a feasible treatment for uncomplicated appendicitis. This article aimed at systematically reviewing the available literatures and discussing the question whether NOM should replace appendectomy as the standard first-line treatment for uncomplicated appendicitis.

**Method:**

A search of the Embase, Pubmed and Cochrane Library was performed using the keywords ‘acute appendicitis’ and ‘antibiotic therapy’. Meta-analysis with inverse variance model for continuous variable and Mantel Haenzel Model for dichotomous variable was performed to evaluate the one year treatment efficacy, morbidities rate, sick leave duration and length of hospital stay associated with emergency appendectomy and NOM.

**Results:**

Six randomized control trials were identified out of 1943 publications. NOM had a significant lower treatment efficacy rate at one year, 0.10 (95% CI 0.03–0.36, *p* < 0.01), when compared to appendectomy. The morbidities rate was comparable between the two interventions. The length of hospital stay was longer, with a mean difference of 1.08 days (95% CI 0.09–2.07, *p* = 0.03), and the sick leave duration was shorter, a mean difference of 3.37 days (95% CI -5.90 to −0.85 days, *p* < 0.01) for NOM.

**Conclusion:**

The paradigm remains unchanged, that appendectomy is the gold standard of treatment for uncomplicated appendicitis, given its higher efficacy rate when compared to NOM.

## Background

Acute appendicitis is one of the commonly encountered acute surgical conditions. Worldwide incidences range from 7.5 to 22.71 per 10,000 and the lifetime risk is around 16.3% [1, 2]. Untreated appendicitis can progress into gangrene or perforation with resultant peritonitis or abscess formation. Since the very first successful appendectomy performed by Claudius Amyand in 1735 [3], it has long been the gold standard of treatment for acute appendicitis. This surgical dogma was first challenged by Fitz in 1886, who suggested that patients with appendicitis may resolve without surgical intervention as evidence of previous appendicitis were found in many autopsy specimens [4]. Coldrey first reported successful treatment of 471 patients with acute appendicitis using antibiotics alone in 1956 [5]. Since then, a number of studies investigated the role of using antibiotics alone, non-operative management (NOM), in the management of acute uncomplicated appendicitis with promising results [6–8]. Appendectomy, albeit a routine surgical procedure with low mortality [9], has a complication rate of 5 to 28% [10]. Given the evidence that supports NOM, should the paradigm of treatment for uncomplicated appendicitis changed from operative to non-operative? This review focused on the current available evidence in the literature comparing NOM and appendectomy for the treatment of acute uncomplicated appendicitis in adults in order to answer this question.

## Methodology

All studies that evaluated the effectiveness of NOM over appendectomy in managing uncomplicated acute appendicitis were retrieved from Medline (PubMed), Embase (1980-) and Cochrane Library electronic databases. The search was carried out on 15th June, 2017. The MeSH term “acute appendicitis” & “antibiotic therapy” were used as search terms. Only two terms were used with the intention to include more literatures for preliminary screening. “antibiotic therapy” was chosen since operative management had been considered as the gold standard for treating appendicitis and trials working on performance of NOM should have compared NOM with the gold standard. It is reasonable to assume using “antibiotic therapy” as search term can identify all trials that compared NOM with appendectomy.

Search mode was set as best matched and “full text” for Pubmed searching. The search terms were used as subject heading for searching in Cochrane and Embase (1980-). Editorials, case reports, expert opinions, letters to the editor, reviews without original data, conference abstract and studies solely on pediatric population were excluded. The screening and selection criteria of studies were summarized in Table 1.

**Table 1:** A summary of the screening and selection criteria of studies

Inclusion Criteria	1. Uncomplicated Acute Appendicitis - Excluded perforation - Excluded intra-abdominal abscess 2. Mainly focus on adult population 3. Full article published in English 4. Randomized control trials & prospective comparative studies
Exclusion	1. Studies that solely involved the pediatric population (Subjects’ age < 18) 2. Studies that only compared elective surgery and conservative management
Search engine	1. PubMed 2. Embase (1980-) 3. Cochrane Library
Keywords	1. Acute appendicitis 2. Antibiotic therapy

### Inclusion criteria

Studies that meet the following criteria were included: 1) Uncomplicated acute appendicitis (Excluding perforation & intra-abdominal abscess); 2) Mainly focused on the adult population; 3) Full article published in English; 4) Randomized control trials (RCT) & observational comparative studies.

### Exclusion criteria

Studies were excluded if they have the following: 1) Studies that solely involved the pediatric population (Subjects’ age < 18); 2) Studies that only compared elective surgery with conservative management.

### Data searches and quality assessments

Embase (1980-), Cochrane Library and Pubmed database were searched by Poon & Wong. The search process was conducted independently and the findings were filled in a preset Excel document. Authors subsequently combined the search result and duplications were removed. Literatures were independently assessed by the authors and then subsequently reviewed together. Consensus was achieved on the inclusion of articles. Quality assessments was performed by Poon and reviewed by Wong.

The primary outcomes that were measured included the success rate, morbidities rate, length of hospital stay and loss of work associated with the two treatment modalities.

### Statistical analysis

Cochrane Review Manager (RevMan) Version 5.3.5 (Copenhagen: The Nordic Cochrane Centre, The Cochrane Collaboration, 2014) was used for evaluating the studies’ results and constructing the forest plots & funnel plots. Odds ratio (OR) of successfully treated cases in patients with NOM when compared to the appendectomy group had been employed to evaluate the outcomes of the two interventions. 95% confidence intervals (95% CI) were decided to evaluate the statistical significance of the OR. An OR of greater than one dictates a superior outcome for the NOM group, and the value of OR was considered statistically significant at the *p* = 0.05 level. Heterogeneity was accounted by the I-square test. Fixed effect model of Mantel Haenzel method was used for analyzing dichotomous data. The analysis of continuous data employed random effect model of inverse variance with regard to the great heterogeneity.

## Results

### Search result

**Fig. 1:**
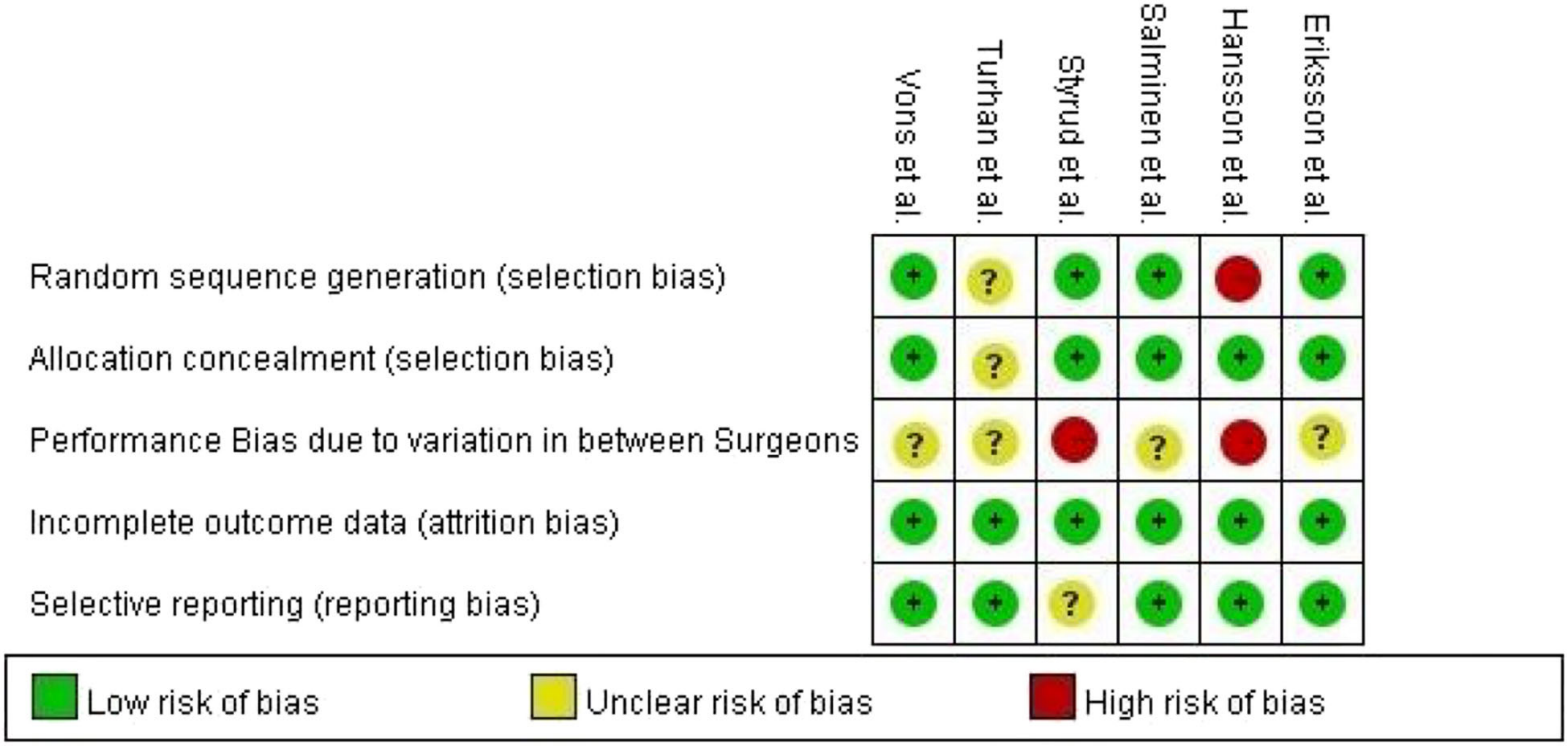
Bias assessment of the included RCTs

In total, 1943 records were identified from the databases (Fig. 1). 1545 were identified from Pubmed, while 369 were identified from Embase. The remaining was identified from Cochrane Library of Systematic Review. After excluding the reviews without original data, letters to editor, case reports, conference abstracts and non-comparative studies, 388 items were left from the three databases. 106 articles were found after screening of titles and abstracts of the 388 items. An addition of six articles were excluded due to duplication. 16 studies were identified for qualitative synthesis. Out of those 16 studies, seven RCTs fulfilling the preset criteria were identified. One RCT was excluded as the article was subsequently retracted due to plagiarism [11]. The included studies were summarized in Table 2.

**Table 2:** A summary of the studies comparing non-operative management and appendectomy

Study, Study Design	Year of publication	Medical Treatment	Surgical Treatment	No. of Patients	Outcome	Remarks
Eriksson et al. [6] Pilot RCT	1995	IV cefotaxime 2 g BID and tinidazole 800 mg daily for 2 days, followed by oral ofloxacin 200 mg BID and tinidazole 500 mg BID for 8 days	Open appendectomy	S:20 + M:20 = T:40	NOM was superior to Appendectomy in pain control NOM had a recurrence rate of 35% in 17 months NOM could effectively manage AUA	CT not applied for diagnosis of appendicitis
Styrud et al. [12] RCT	2006	IV cefotaxime for 2 days and tinidazole 800 mg daily, followed by ofloxacin 200 mg	Open or laparoscopic approach on surgeon preference	S:124 + M:128 = T: 252	Appendectomy had a higher complication rate NOM could successfully manage AUA	Female patients excluded
Hansson et alia [13] RCT	2009	IV cefotaxime 1 g BID and metronidazole 1·5 g q24hr for 1 day, followed by oral ciprofloxacin 500 mg BID and metronidazole 400 mg TID for 10 days	Open or laparoscopic approach as surgeons’ usual practice	S:167 + M:202 = T:369	NOM was safe in AUA NOM had a recurrence rate of 13.9% in 1 year Appendectomy was 3 folds higher in major complication rate Minor complication rate was similar	This study has a high cross over rate from NOM to appendectomy.
Malik et al. [11] RCT	2009	IV ciprofloxacin 500 mg BID and metronidazole 500 mg TID for 2 days, followed by oral ciprofloxacin 500 mg BID and tinidazole 600 mg BID for 7 days	Approach not specified	S:40 + M:40 = T:80	NOM was superior to Appendectomy in pain control NOM was superior in lowering white cell count and temperature in early course NOM had more recurrence	This article was retracted due to plagiarism.
Turhan et al. [14] RCT	2009	IV ampicillin 1 g QID, gentamicin 160 mg daily and metronidazole 500 mg TID for 3 days, followed by oral antibiotics for 10 days	Open or laparoscopic appendectomy	S:183 + M:107 = T: 290	Appendectomy was superior to NOM in length of hospital stay NOM cost less than Appendectomy No difference in morbidity	
Vons et al. [15] RCT	2011	Amoxicillin and clavulanic Acid of 3 g or 4 g according to weight, with route and duration according to clinical symptoms	Open or laparoscopic approach on surgeon preference	S:123 + M:120 = T: 243	Appendectomy was superior to NOM	
Salminen et al. [19] RCT	2015	IV ertapenem 1 g daily for 3 days, followed by oral levofloxacin 500 mg daily and metronidazole 500 mg TID for 7 days	Open appendectomy	S:273 + M:257 = T: 530	Appendectomy had a higher overall complication rate than NOM NOM had longer hospital stay NOM failed to demonstrate non-inferiority No significant difference in treatment efficacy	

S = Number of surgically managed patients; M = Number of medically managed patients T = Total number of patients involved in the study

NOM = Non-operative management; AUA = Acute uncomplicated appendicitis

Superior refers to statistically significant in clinical outcome by parameter used by the authors of the respective studies

### Critical appraisal of the studies

**Fig. 2:**
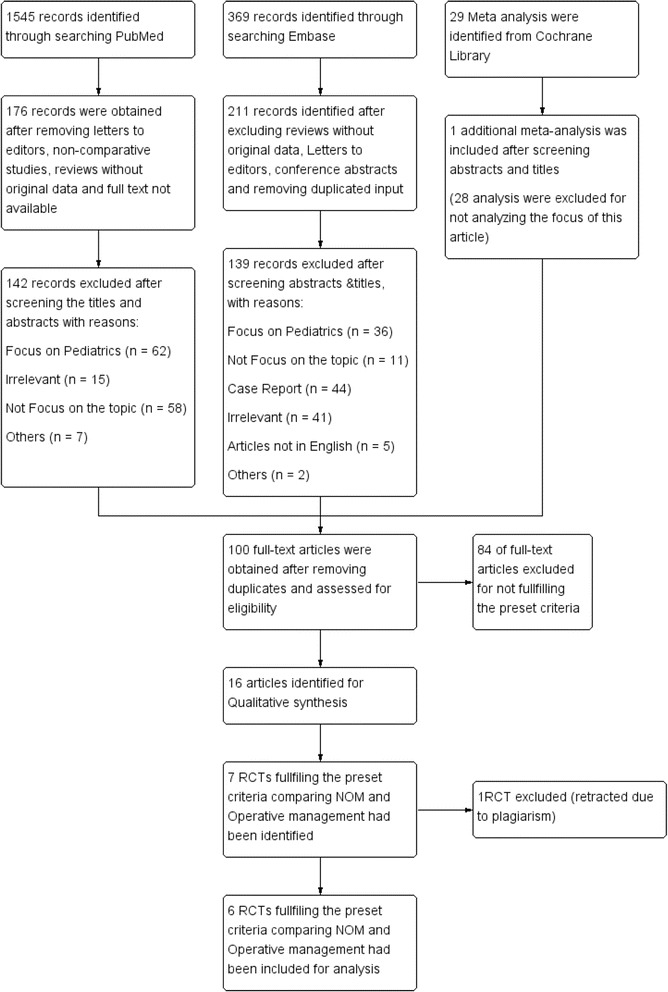
PRISMA flow diagram

Figure 2 summarized the bias assessment of the included studies. Eriksson et al. [6] was the first randomized trial that compared the outcome of NOM with appendectomy. The study was limited by small sample size, with only 40 patients recruited. The diagnoses were based on clinical signs and ultrasonographic findings and none had a computer tomography (CT) scan prior to surgery. Diagnostic inaccuracy is one of the main pitfalls of many studies published in the literature as some of the recruited patients were indeed suffering from complicated appendicitis. There is a chance of underestimating the efficacy of NOM in uncomplicated appendicitis and partly explains the high recurrence rate of 35% after 7 months of follow-up in the NOM group.

Styrud et al. conducted the first large scale randomized trial and it was the follow-up RCT of Eriksson’s pilot study [12]. It reported a failure rate of NOM up to 12% at 24 h after the initiation of treatment. Among those patients, 47% of them were subsequently found to have perforated appendicitis. In this study, the randomization process and demographics of the excluded patients were clearly stated. However only male subjects and patients younger than 50 were included in view of ethical concerns from the local ethics committee. This limited its ability to address the role of NOM in elderly patients. The diagnosis of uncomplicated acute appendicitis was made on clinical grounds alone, without the use of imaging studies. There was a relatively high post-operative complication rate in the appendectomy group, 14%, mainly due to wound infection. It was difficult to generalize its results in the era of minimally invasive surgery as the author indiscriminately included both laparoscopic and open approaches.

Hansson et al. published their study in 2009 [13]. The diagnosis of uncomplicated appendicitis was based on clinical grounds with or without the use of imaging studies. This study adopted an unclassical method of subject randomization; using the date of birth of patients, with uneven date being the NOM group. This resulted in a huge difference in the number of subjects among the two groups and resulted in high selection bias. Furthermore, the study had a high crossover rate from the NOM arm to the appendectomy arm. Nearly half of the patients (96 out of 202) assigned to NOM were crossed over to surgery because of either surgeons’ preference or patients’ request. Besides, only less than half of the patients in the NOM group were followed up one year later. The rate of recurrence in patients treated with NOM was therefore likely underestimated. The study suggested that NOM was a safe alternative to appendectomy. However, the aforementioned factors made the trial less reliable.

Turhan et al. published their result in 2009 [14]. This study specified itself as a randomized control trial. Nevertheless, the randomization process was not clearly stated. This might result in potential bias. The diagnosis of uncomplicated acute appendicitis was confirmed by either ultrasonography or computed tomography.

The study published by Vons et al. involved patients from six hospitals [15]. Vons et al. used clearly defined diagnostic criteria with the aid of high quality CT scan to confirm the diagnoses in the study. Despite this, there was still an incidence of 18% for perforated appendicitis on upfront surgery. Randomization method and the results of the trials were well documented. The study also evaluated the risk factors associated with the absence of improvement of appendicitis in the NOM group. Despite no multivariate regression performed, fever at initial presentation, high presenting serum C-reactive protein level and intraluminal fecolith, were likely predictors for poor response to NOM. The antibiotics of choice in the NOM group in this study was amoxicillin and clavulanic acid, with an initial failure rate of 11.7%, which was relatively high compared to other studies. This might be explained by the fact that, *Escherichia coli*, being the most common bacterial isolates from patients with acute appendicitis, have a high percentage of resistance to amoxicillin and clavulanic acid [16, 17]. This regime is currently not recommended for the treatment of acute appendicitis [18].

The study performed by Salminen et al. is the largest available RCT focusing on this topic to date [19]. It included 530 patients recruited in Finland. The study documented well the reason for excluding patients from the trial. Other components including the process of randomization, standardization between different hospitals and diagnostic criteria were clearly stated. These found the reliability of the results of this trial. Ertapenem was the antibiotics of choice in this study. It was preferred due to its efficacy as a single agent for abdominal infections [19]. The initial failure rate was 5.7%. Despite the fact that it demonstrated a significant shorter hospital study for the operated group, the postoperative sick leave period was significantly longer in the appendectomy group. The study failed to demonstrate non-inferiority for NOM over appendectomy in terms of treatment efficacy. Nevertheless, the setting of a 24% non-inferiority margin was challenged [20].

### Treatment success rate

Treatment success was defined as resolution of appendicitis without the development of complications or recurrence necessitating interventions. The majority of the comparative studies favored appendectomy in this regard. Hasson et al. demonstrated a 48% one-year success rate for NOM, compared to 89.2% for appendectomy [13]. Salminen et al. reported a higher success rate of 70.4% for NOM [19]. Similar result was published by Vons et al., where the successful rate of NOM was 67.5% [15]. The same study also demonstrated that the presence of fecolith on CT scan, was associated with higher chance of failure for NOM (50% vs 14%). Overall, the recurrence rate ranged from 13.9 to 35% in the first year [6, 12, 13, 15, 19].

Pooled analysis of one year treatment efficacy was performed by the random effect model of Mantel Haenzel method with respect to the high heterogeneity (Fig. 3.3). The sample sizes of patients were included in the above analysis. It demonstrated a significant advantage in surgically managed patients when compared to those with NOM. The odds ratio for successful treatment in the NOM group at one year was only 0.10 (95% CI 0.03–0.36, *p* < 0.01), when compared with the surgically managed group. Sensitivity test, which remained in favor of surgical management, was performed by alternatively removing the largest and smallest study. Both tests resulted in significant advantages for the operated group.

**Fig. 3:**
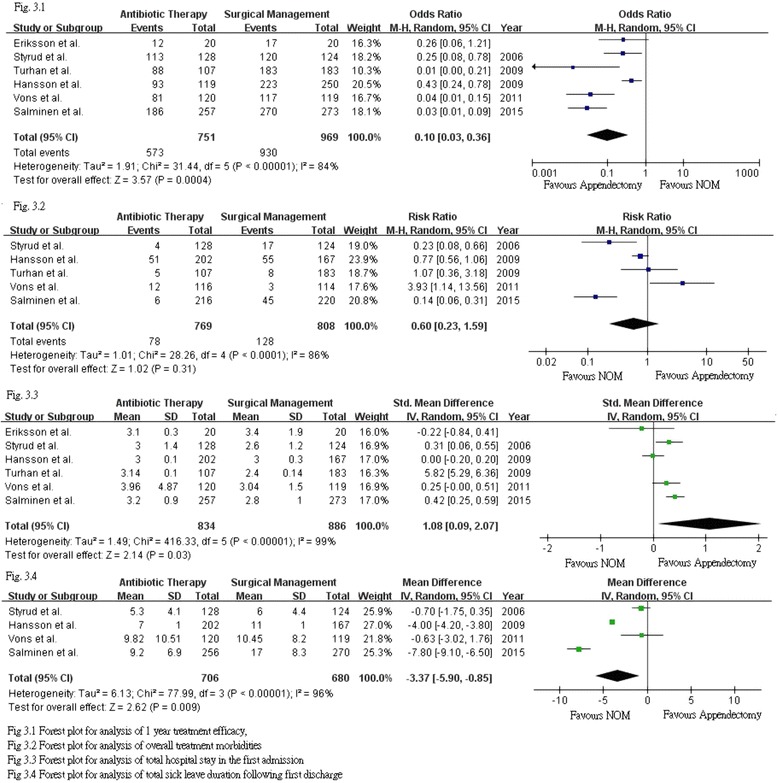
Forest plots of meta-analysis results

### Morbidities

Except the one from Eriksson et al..., all studies provided the incidence of overall complications following each intervention. The results from Turhan et al. & Vons et al*’s* studies supported appendectomy while the remaining three studies favored NOM. Analysis was performed with Mantel Haenszel random effect model. The overall complication rate after excluding recurrence was compared. The analysis demonstrated a comparable result between appendectomy and NOM with a risk ratio of 0.60 (95%CI: 0.23–1.59, *p* = 0.31), favoring NOM. The majority of these complications were related to infections. A high heterogeneity was noted with regard to the results. It might be accounted by the difference in antibiotic regimes and the surgical approaches used. Sensitivity tests were performed by excluding the largest and smallest studies alternatively. The complication rate remained comparable between the two interventions. Separate analysis for major and minor complications was not performed as only Hasson et al. clearly listed the nature of complications.

### Length of hospital stay

Pooled analysis of the length of stay for the initial admission demonstrated a significant longer hospital stay in the NOM group. A total of 1720 patients were included in the analysis. Random effect model was employed with regard to the high heterogeneity in the studies included. The standardized mean difference in hospital stay after pooled analysis was 1.08 (95% CI 0.09–2.07, *p* = 0.03). Sensitivity test was performed by removing the largest and smallest study respectively. A significant advantage was still noted after removing the smallest scaled study by Eriksson et al. [6]. The significance disappeared after removing the study by Salminen et al. [19].

### Loss of work

The duration of sick leave was significantly shorter in the NOM group, with a mean difference of 7.8 days (95% CI 6.5 to 9.1) in Salminen’s study [19]. This is in line with the pooled analysis of four RCTs in this review. The analysis of the total sick leave duration after the first admission included a sample size of 1380. Random effect model was employed with regard to the high heterogeneity in the studies included. Significance was detected between the two intervention groups with a trend favoring NOM. The mean difference noted from the analysis was 3.37 days less for the NOM group with 95% CI of −5.90 to −0.85 day, *p* value <0.01 was noted. Sensitivity test was not performed as only 4 studies were included for analysis.

## Discussion

This review demonstrated a higher efficacy for appendectomy and was consistent with earlier meta-analysis [21, 22]. In the meta-analysis by Sallinen et al., including five RCTs, one on pediatric patients, 8.5% of the patients treated with NOM required appendectomy within the first month [23]. Wilms et al. published a meta-analysis involving five RCTs and 901 patients [24]. Although 73.4% of the patients treated with NOM had resolution of acute appendicitis without major complications and recurrence in the following year, as compared to 97.4% in the appendectomy group, the author could not demonstrate non-inferiority of NOM and concluded that more than one out of five patients treated with NOM would develop either complications or recurrence requiring surgery in the following year. A consensus statement was issued by the expert panel at the 3rd World Congress of the World Society of Emergency Surgery held in Jerusalem, Israel in 2015, which stated that NOM could be successful in selected patients with uncomplicated appendicitis who wish to avoid surgery and accept a higher risk, up to 38%, of recurrence [25].

The complications rate of appendectomy was generally higher than NOM in the literature. In Mason’s meta-analysis [26], NOM was shown to be protective for major and minor complications with an odds ratio of 0.54 (95% CI 0.37 to 0.78). This is contrary to results from some non-randomized studies. Data from a national database involving the treatment of 436,400 cases of acute appendicitis over a 8-year period showed a significantly higher rate of in-hospital complications (27.8% vs. 7%, *p* < 0.001) and longer hospital stay (3 vs. 2 days, p < 0.001) in the NOM group [27]. It is unfortunate that all RCTs comparing NOM and surgery, except one [15], had a high rate of open appendectomy, ranging from 82 to 100% [6, 12, 14, 19]. Further, the choice of operative approach was not standardized and was often up to surgeons’ discretion. It is difficult to generalize these results in terms of morbidities from the available RCTs as the majority of patients, up to over 90%, with acute appendicitis were operated with the laparoscopic approach nowadays [28]. Previous RCTs and systematic reviews have shown that laparoscopic approach was associated with fewer wound complications, less postoperative pain and shorter hospital stay [29] but with a higher incidence of intra-abdominal collection [30].

The results of the earlier RCTs did not show a difference between NOM and surgery in terms of hospital stay [6, 12, 13]. The two latest RCTs published in 2011 and 2015 showed shorter hospital stay in the appendectomy group [15, 19]. A meta-analysis showed shorter hospital stay in the appendectomy group, with a mean difference of 0.41 days (95% CI 0.26 to 0.57) [23]. This is in line with the result of this review, which the surgically treated patients had a shorter hospital stay than those with NOM. Nevertheless, two other meta-analysis failed to demonstrate a significant difference in hospital stay when only adult RCTs were included [21, 22]. As mentioned above, the percentage of patients operated with the laparoscopic approach in the RCTs was far less than expected. Shorted hospital stay and faster return to work would be expected in laparoscopic appendectomy. On the other hand, the use of oral antibiotics in NOM, as demonstrated in the Non Operative Treatment for Acute Appendicitis (NOTA) study conducted in Italy, would largely reduce the length of hospital stay [31]. It is difficult to draw a conclusion whether NOM has an advantage over appendectomy based on the current evidence.

In terms of long term efficacy, results beyond one year from the published RCTs were not available in the literature. A retrospective study on 3236 patients treated with NOM revealed a long-term recurrence rate of 4.4% at a mean follow up of 7 ± 3.9 years [32]. Another study on 118 patients treated with NOM showed a 10.2% recurrence at a median follow-up of 23 months [33]. The NOTA study suggested a long term efficacy of 83% up to two years with NOM [34].

Given the aforementioned one-year recurrence rate of 13.9 to 35%, the majority of the patients primarily treated with NOM would remain symptoms-free. Routine interval appendectomy after resolution of symptoms was therefore not necessary. On the other hand, the morbidities rate of interval appendectomy was low. In the study by Salminen, none of the patients suffered from intra-abdominal abscess after interval appendectomy [19]. The surgical complication rate of elective interval appendectomy was lower than primary appendectomy by 13.4%.

Apart from a higher treatment efficacy rate, appendectomy offers definitive histology and provides a chance to diagnose rare appendiceal and extra-appendiceal pathologies. Connor reported the presence of appendiceal tumors in 0.9% of 7970 appendectomy specimens [35]. Another study reported a 2.7% and 1.1% incidence of appendiceal diverticulitis and appendiceal tumors, which included carcinoid tumors, adenocarcinoma, mucinous cystadenoma etc. [36].

## Conclusion

NOM is definitely a feasible and effective alternative for uncomplicated appendicitis. The majority of patients primarily treated with NOM would be spared from post-operative pain, surgical risks and wound complications. The paradigm remains unchanged, however, that appendectomy should be the gold standard of treatment given its higher treatment success rate and shorter hospital stay. Current evidence in the literature mainly focused on comparing NOM with open appendectomy. With widespread adoption of the laparoscopic approach, high quality evidence is still needed in the comparison of primary laparoscopic appendectomy and NOM. Studies evaluating factors that could affect the rate of success in NOM is needed for clinical reference and to tailor treatment on an individual basis. Until then, patients should be well informed of the available treatment options, their pros and cons, so as to make informed decision and benefit from the optimal treatment.
